# Training hospital managers for strategic planning and management: a prospective study

**DOI:** 10.1186/s12909-015-0310-9

**Published:** 2015-02-26

**Authors:** Zorica Terzic-Supic, Vesna Bjegovic-Mikanovic, Dejana Vukovic, Milena Santric-Milicevic, Jelena Marinkovic, Vladimir Vasic, Ulrich Laaser

**Affiliations:** 1 Institute of Social Medicine, University of Belgrade, School of Medicine, Belgrade, Serbia; 2 Centre-School of Public Health and Management, University of Belgrade, School of Medicine, Belgrade, Serbia; 3 Institute of Medical Statistics and Informatics, University of Belgrade, School of Medicine, Belgrade, Serbia; 4 Department of Statistics and Mathematics, Faculty of Economics, University of Belgrade, Belgrade, Serbia; 5 Section of International Public Health (S-IPH), Faculty of Health Sciences, University of Bielefeld, Bielefeld, Germany

**Keywords:** Training, Learning outcome, Hospital management, Strategic planning, Professional development

## Abstract

**Background:**

Training is the systematic acquisition of skills, rules, concepts, or attitudes and is one of the most important components in any organization’s strategy. There is increasing demand for formal and informal training programs especially for physicians in leadership positions. This study determined the learning outcomes after a specific training program for hospital management teams.

**Methods:**

The study was conducted during 2006 and 2007 at the Centre School of Public Health and Management, Faculty of Medicine, University of Belgrade and included 107 participants involved in the management in 20 Serbian general hospitals. The management teams were multidisciplinary, consisting of five members on average: the director of the general hospital, the deputy directors, the head nurse, and the chiefs of support services. The managers attended a training program, which comprised four modules addressing specific topics. Three reviewers independently evaluated the level of management skills at the beginning and 12 months after the training program. Principal component analysis and subsequent stepwise multiple linear regression analysis were performed to determine predictors of learning outcomes.

**Results:**

The quality of the SWOT (strengths, weaknesses, opportunities and threats) analyses performed by the trainees improved with differences between 0.35 and 0.49 on a Likert scale (*p* < 0.001). Principal component analysis explained 81% of the variance affecting their quality of strategic planning. Following the training program, the external environment, strategic positioning, and quality of care were predictors of learning outcomes. The four regression models used showed that the training program had positive effects (*p* < 0.001) on the ability to formulate a Strategic Plan comprising the hospital mission, vision, strategic objectives, and action plan.

**Conclusion:**

This study provided evidence that training for strategic planning and management enhanced the strategic decision-making of hospital management teams, which is a requirement for hospitals in an increasingly competitive, complex and challenging context. For the first time, half of state general hospitals involved in team training have formulated the development of an official strategic plan. The positive effects of the formal training program justify additional investment in future education and training.

## Background

Strategic management comprises drafting, implementing, and evaluating cross-functional decisions that enable an organization to achieve its long-term strategic objectives [1]. Changes in the external environment (i.e., demographic and epidemiological transitions, economic fluctuations, public and political expectations), and within the health care system (i.e., health market, demands, costs, new technologies, regulations) have put pressure on hospital managers to implement strategic management programs to respond to environmental challenges [2-5]. Leadership development [6] implies that a Strategic Plan should be in place, with the organization’s mission, vision, strategic objectives, and action plans aimed at achieving these objectives [7]. A hospital develops strategies, which must be derived from a situational or strategic analysis, which most often is performed by a SWOT analysis (strengths, weaknesses, opportunities and threats).

Continuing education and training have become part of the ongoing processes of organizational learning and permanent change, employee evaluation, and career development [8]. In addition, they are essential tools for managers to improve their management skills and to learn new skills [9-11]. Training can be defined as the systematic acquisition of skills, rules, concepts, or attitudes, which result in improved performance [12]. There is an increasing demand for formal and informal training programs in health organizations, especially for physicians in leadership positions who need to acquire managerial and leadership skills. In some hospitals, significant resources are devoted to educate the institution’s managers [13]. Training is one of the most important components in any organization’s strategy, and evaluation is an essential part of the training system [14].

However, organizations, including hospitals, spend little time on evaluating their training programs [15-20]. Numerous reasons have been identified: disbelief in the benefits of evaluation, lack of confidence in whether training has an impact on the organization, as well as lack of resources, expertise, and organizational support [21]. The expansion of Kirkpatrick’s four-level evaluation model has emphasized the importance of training evaluation [15]. This model includes measurement of reactions to training, learning attainment, transfer and subsequent individual behavior, and organizational results [16]. Most evaluations of this model have focused on its first two levels. Several authors have also researched the effect of training on individual job performance or the results achieved within their own organization [17-19]. The benefits of training evaluation are widely recognized as a diagnostic tool for training revisions, and for evidence of training effectiveness, such as successful learning, improved on-the-job performance, changes in key business measures, and return on investment. In addition, training evaluation should influence decision making [22-24]. Furthermore, training evaluation may have ethical implications for professional development [25].

In Serbia, the health care system has declined during the past two decades due to the country’s unstable political situation and long-term, weak economic development [25]. Consequently, in many cases the performance of Serbian hospitals has yet to reach a satisfactory level [25]. Therefore, there is a demand for systematic interventions, including multidisciplinary management training for stronger management of the health care system in Serbia. This was recognized by different international organizations and supporting management capacity building programs [26,27]. Formal training of health managers was not common in Serbia before the democratic transition that took place in the year 2000 [28]. In the mid - 2000, the Serbian government adopted laws which supported formal education and the necessity to improve the specific skills and knowledge of health professionals in the field of health care management (continuing education, postgraduate academic programs) [29-31]. The recognition and application of advanced managerial skills became basic requirements for managing hospitals as well as other health care institutions in Serbia. This has influenced the quality of services, accountability and effectiveness in the hospitals [32,33].

An earlier study focused on the skills improvement of individual hospital managers [34]. The aim of this study was to determine the learning outcomes after a specific training program for hospital management teams.

## Methods

### Study design

The study was conducted during 2006 and 2007 at the Centre School of Public Health and Management, Faculty of Medicine, University of Belgrade. Twenty management teams from 40 general hospitals in Serbia took part. Hospitals eligible for training were selected by the Ministry of Health of Serbia and participated in the capacity-building project sponsored by the Ministry and the European Agency for Reconstruction [35]. The participating hospitals had an average number of approximately 300 beds.

The management teams were multidisciplinary, consisting of five members on average: the director of the general hospital, the deputy directors, the head nurse, and the chiefs of support services. The total number of managers was 107. Managerial teams comprised clinical physicians (27.1%), economists (21.5%), lawyers (18.7%), engineers (16.8%), and nurses (14.0%). The specialists among the physicians were mostly from surgery (24.3%) and internal medicine (12.6%). The average age of the managers was 47.7 ± 7.0 years. The average number of years of service was 21.9 ± 7.8 and for managerial experience 11.1 ± 8.0 years. The share of female managers who held the position of director or deputy director was 30.6%. Almost two-thirds (63.6%) of the participating managers had previously attended some form of managerial training (seminars, workshops), but without obtaining a formal management degree or certificate.

### Description of training program

During a six-month period, the hospital management teams attended training for health management that comprised four sessions of 48-hour modules addressing specific topics: Basic Health Management, Hospital Management, Health Information Management, and Total Quality Management. Twenty hours were contact hours in the classroom and 40 included individual work, which involved 60 learning hours per module.

The module of Basic Health Management included basic functions of management, the specifics of management in health care organizations, and the application of strategic management tools, such as SWOT and Strategic Plan development (the mission, vision, objectives, and action plan) [36,37] (Table 1).

**Table 1:** **Examples of a vision and mission statement and goals**

**Vision statement**	“Leader position among the hospitals in Serbia; establishing new treatment standards of our patients“.
**Mission statement**	“We are here to provide optimal methods in health care services with respect for the demands of our patients and to apply new technological accomplishments for the faster and more efficient treatment of our customers“.
**Goals**	• Development of quality and efficiency of health care services
	• Establishing new diagnostic and therapeutic methods
	• Implementation of procedures for ambulatory surgery

During the module of Basic Health Management, each participating management team performed the first SWOT analysis of their hospital. Throughout the entire six-month training period, the management teams were able to consult with trainers, either by e-mail or directly, regarding their work in the context of preparing the second SWOT analysis and developing a Strategic Plan for the hospital.

Based on identification of the strengths, weaknesses (Table 2), opportunities, and threats (Table 3), the teams attempted to agree on the best of the four SWOT strategies [36-39] for their own hospital, listed below (Table 4).

**Table 2:** **The examples for internal strengths and weaknesses were related to the following categories: employees, activities, technical resources, customers, finance, management**

**Examples of strengths**	• Highly educated staff
	• Introduction of clinical guidelines
	• Renovation of some parts of our facilities
	• Good relationship with the media
**Examples of weaknesses**	• Medical staff holding second jobs in private practice
	• Medical equipment out of date
	• Low motivation of staff
	• Negative financial balance

**Table 3:** **Examples of external opportunities and threats were related to the following categories: legislation, demographic characteristics of the population, economy, the political situation, market**

**Examples of opportunities**	• Rationing of hospital staff and facilities
	• Support from the local community and NGOs
	• Participation in international projects
**Examples of threats**	• Lack of treatment standards and protocols
	• High number of refugees and internally displaced people
	• Lack of effective gatekeeper function in primary health care

**Table 4:** **SWOT analysis – strategies**

**Comparative advantage (Strength/Opportunity)**	Widen the spectrum of services to gain additional income
**Investment/Divestment (Weakness/Opportunity)**	Promotion of cooperation with local authorities
**Mobilization (Strength/Threat)**	Improvement of communication with customers
**Damage control (Weakness/Threat)**	Note: The teams could not or did not want to imagine this scenario

*Comparative Advantage Strategy or Maxi-Maxi* means the ability of hospitals to maximize strengths and opportunities and take advantage of the market. *Investment/Divestment Strategy or Mini-Maxi* means to minimize the weaknesses and to maximize the opportunities. The question is, should a hospital invest its scarce resources in weak programs in order to make them more competitive vis-à-vis a perceived opportunity? *Mobilization Strategy or Maxi-Mini* is based on the strengths of the hospital that can deal with the threats of the environment and the possibility of mobilizing its strengths to avert a perceived threat or even transform this threat into an opportunity. *Damage Control Strategy or Mini-Mini* is a survival strategy, which includes temporary measures to minimize weaknesses of, and threats to, a hospital (Table 4).

The Hospital Management module aimed to develop management and leadership capacity for hospital management, using the basic principles of social marketing and also to assess hospital performance, payment, and organizational modalities. The third module, Health Information Management, explored information flow in the health care system, computer-assisted learning, and document-management systems. Finally, within the fourth module, Total Quality Management (TQM), managers learned about quality in health care, analysis of quality indicators, and the application of the TQM method.

Twelve months after the beginning of the training program, within the Change Management module, the hospital management teams presented a Strategic Plan for their hospital based on the second SWOT analysis. The managers had been prepared for this task throughout the four modules by a combination of lectures, case studies, task-orientated activities, and directed learning.

### Evaluation procedure

Three independent reviewers of the training program, from the university environment (Faculty of Organizational Sciences and Faculty of Medicine, Departments of Epidemiology and of Social Medicine) performed a review after the end of the training program. Two reviewers had a PhD degree in Management, while the third one had certificates from several relevant international training centres in the field of Health Management. All three reviewers had experience and were involved in health care system work. The average length of managerial experience was 18 ± 1.63 years. They assessed the quality of the two anonymous SWOT analyses carried out by the 20 management teams: the first SWOT was delivered at the beginning of training during the first module (20 analyses of SWOT-I), and the second SWOT, 12 months later (20 analyses of SWOT-II). They also assessed the quality of the Strategic Plans (20 hospital plans) developed by the teams after the second SWOT analysis. The reviewers did not know in advance which hospital SWOT analyses or Strategic Plans they would evaluate. Each reviewer assessed 40 SWOT analyses (SWOT-I and SWOT-II) and 20 hospital strategic plans.

The reviewers used a specific questionnaire comprising 37 questions, organized in four parts following the hospital’s mission and vision and its objectives and action plan [36,39]. The answers to the questionnaire were ranked on a Likert scale from 1 (very poor) to 5 (excellent). Questions relating to the quality of SWOT analysis were directed at the analysis of the external and internal environment and how technical rules were applied in the SWOT analyses. For example, the number of items pertaining to the external and internal environment, whether the items were arranged from most to least important and according to the external and internal environment, and identification and clarification of hospital strategies.

Next, questions regarding the managers’ vision statements focused on the hospital’s state and function and whether the vision was culture-specific, brief, verifiable, focused, flexible, understandable to all employees, and inspirational. Questions relating to the mission statement addressed the organization’s purpose, its business values, client orientation, and use of proactive verbs to describe what hospitals do. The evaluation of strategic objectives considered the number of objectives and whether they were SMART (i.e. specific, measurable, achievable, relevant, and time frame) [39]. Finally, the action plan was evaluated in relation to activities, results, staff responsibility, realistic budgeting, and the existence of a time frame for each objective.

### Statistical analysis

The continuous variables, according to the Likert scale, were expressed as means and their standard deviation. If the group variances were not homogenous, as evidenced by Levene’s test**,** the *p*-values were adjusted. The internal consistency and reliability of each reviewer were determined using intra-class correlation coefficients (ICC) related to the means. The significance of the ICCs was tested by a two**-**way ANOVA random effects model. Cronbach's alpha was used to estimate the internal consistency of questions that comprised several items, such as those regarding technical skills in the SWOT analysis, SMART objectives, and action plan. The internal consistency was considered acceptable when Cronbach’s alpha coefficient ≥0.7 [40]. Paired sample tests were used to compare means and thus identify differences indicative of improvement for items of the SWOT analyses conducted at the beginning of the program and again 12 months later. Principal component (PC) analysis with varimax rotation and the Kaiser criterion was applied with 13 variables in the SWOT analyses, before and after training, to detect common dimensions. Principal components were described by variables with a loading > 0.7. A stepwise multiple linear regression analysis was applied to determine predictors of quality of the Strategic Plans developed by the managerial teams (hospital mission, vision, strategic SMART objectives, and action plan).

### Ethical approval

The study was reviewed and given ethical approval by the Ethics Committees at the School of Medicine in Belgrade. All participants received cover letters which contained detailed information on objectives and methodology of the research and consent forms. All participants gave written, informed consent.

## Results

The consistency of judgments given by each reviewer for the SWOT analyses ranged between 0.50 and 1.00 (Table 5) and for the Strategic Plans between 0.92 and 0.97 (Table 6). There was a high degree of matching among the reviewers, especially in the evaluation of the Strategic Plan (*p* < 0.001 for the intra-class correlation coefficient), assessed twelve months after the end of the training program (Table 6).

**Table 5:** **Experts’ evaluation of SWOT analyses at the beginning of the training program and 12 months after**

SWOT analysis	ICC ^ †† ^		Evaluators’ grading		Differences*
			Before (SWOT I)	After (SWOT II)	
	Before	After	Mean ± SD	Mean ± SD	Mean ± SD
External environment:					
Analysis of macro-environment	0.84	0.83	3.19 ± 0.67	3.68 ± 0.65	0.49 ± 0.19^††^
Evidence-based analysis of environment	0.75	0.73	2.87 ± 0.51	3.29 ± 0.53	0.43 ± 0.18^††^
Analysis of stakeholders	0.80	0.64	2.98 ± 0.66	3.46 ± 0.65	0.48 ± 0.22^††^
Analysis of competitors	0.83	0.76	2.89 ± 0.57	3.33 ± 0.53	0.44 ± 0.24^††^
External analysis relates to mission & vision	0.67	0.61	3.07 ± 0.69	3.44 ± 0.71	0.37 ± 0.17^††^
Internal environment:					
Internal analysis	0.71	0.73	3.23 ± 0.56	3.64 ± 0.63	0.41 ± 0.21^††^
Analysis of customers	0.67	0.78	2.87 ± 0.64	3.26 ± 0.61	0.38 ± 0.33^††^
Analysis of general resources	0.50	0.96	3.31 ± 0.53	3.71 ± 0.46	0.40 ± 0.25^††^
Analysis of services	0.78	0.71	3.19 ± 0.44	3.54 ± 0.43	0.35 ± 0.22^††^
Analysis of financial resources	0.92	1.00	3.28 ± 0.62	3.75 ± 0.63	0.47 ± 0.25^††^
Hospital performance	1.00	0.95	3.54 ± 0.53	3.97 ± 0.48	0.42 ± 0.25^††^
Technical skills in application of SWOT analysis	0.88**	0.89**	3.22 ± 0.47	3.65 ± 0.45	0.44 ± 0.14^††^
Number of items in the external and internal environment	0.93	0.92	3.46 ± 0.45	3.86 ± 0.46	0.39 ± 0.19^††^
Items are arranged from most to least important	0.93	0.96	3.16 ± 0.59	3.62 ± 0.56	0.46 ± 0.22^††^
Items are arranged according to external and internal environment	0.96	0.95	3.02 ± 0.53	3.48 ± 0.47	0.46 ± 0.23^††^
Identification of strategies	0.97	0.95	3.54 ± 0.46	3.98 ± 0.40	0.45 ± 0.24^††^

*mean difference = after – before.

**α = Cronbach’s alpha coefficient for internal consistency of questions which comprised several items (recommended value ≥ 0.70).

ICC = intraclass correlation coefficient.

^††^*p* < 0.001.

**Table 6:** **Experts’ evaluation of strategic plan after the training program**

Strategic plan	ICC ^ †† ^	Mean ± SD
Hospital’s mission	0.96	4.02 ± 0.91
Hospital’s vision	0.92	4.19 ± 0.79
Strategic SMART objectives: α = 0.97		3.62 ± 1.08
Specific	0.95	3.62 ± 1.19
Measurable	0.93	3.36 ± 1.01
Achievable	0.92	3.79 ± 1.06
Relevant	0.93	3.77 ± 1.03
Time frame	0.96	3.57 ± 1.38
Action plan: α = 0.96		3.44 ± 1.14
Well-defined activities	0.96	3.37 ± 1.22
Well-defined results	0.95	3.39 ± 1.22
Identification of responsible staff	0.97	3.45 ± 1.30
Realistic budgeting	0.95	3.30 ± 1.10
Existence of a time frame	0.96	3.69 ± 1.28

α = Cronbach’s alpha coefficient for internal consistency (recommended value ≥ 0.70).

ICC = intraclass correlation coefficient.

^††^*p* < 0.001.

In the first SWOT analysis, the reviewers gave the lowest grades for the analysis of several external factors (evidence-based analysis of the environment, analysis of competitors and stakeholders). However, all grades improved significantly in the second SWOT analysis after the training program (*p* < 0.001). With regard to the Strategic Plan, the three reviewers assessed the learning outcomes with grades ranging from 3.30 (realistic budgeting) to 4.19 (hospital vision).

In a PC analysis of the 13 SWOT variables, two components (PC I) before training and three components (PC II) after training together explained 70.2% and 80.9%, respectively, of the variance affecting strategic planning. Prior to training, the first principal component was strategic planning ability (PC I-1); the second was internal environment (PC I-2). After training, the principal components were: external environment (PC II-1), strategic positioning (PC II-2), and quality of care (PC II-3) (Table 7). These new variables, i.e., the principal components, were used as independent variables in the subsequent regression analysis to determine predictors of learning outcomes (Table 8).

**Table 7:** **Principal component (PC) analysis of SWOT variables affecting strategic planning (PC I for SWOT-I and PC II for SWOT-II)**

Variables of SWOT analysis	Before training - SWOT-I		After training – SWOT-II		
	Strategic planning ability - PC I-1	Internal environment – PC I-2	External environment – PC II-1	Strategic positioning – PC II-2	Quality of care – PC II-3
Analysis of macro-environment	0.56	0.56	0.55	0.31	0.57
Evidence-based analysis of environment	0.61	0.46	0.85	0.16	0.25
Analysis of stakeholders	0.84	0.29	0.81	0.41	0.16
Analysis of competitors	0.68	0.47	0.75	0.41	0.25
External analysis relates to mission & vision	0.43	0.68	0.68	0.03	0.57
Internal analysis	0.53	0.75	0.63	0.15	0.61
Analysis of customers	0.12	0.84	0.06	0.22	0.85
Analysis of general resources	0.28	0.86	0.25	0.26	0.80
Analysis of services	0.77	0.19	0.20	0.75	0.35
Analysis of financial resources	0.73	0.36	0.79	0.49	0.17
Hospital performance	0.84	0.35	0.74	0.56	0.08
Technical skills in application of SWOT analysis	0.87	0.22	0.50	0.77	0.24
Identification of strategies	0.65	0.27	0.23	0.84	0.17
Variance (%)	41.60	28.62	36.29	23.16	21.46

**Table 8:** **Stepwise multiple linear regression models of hospital mission, vision, SMART objectives, and action plan using the independent components**

Model	Before training					After training				
Dependent variable	Independent components	B	p	95% CI for B		Independent components	B	p	95% CI for B	
				Lower	Upper				Lower	Upper
Hospital mission	Constant = 4.03					Constant = 4.03				
	Internal environment	0.55	0.000	0.44	0.66	Quality of care	0.52	0.000	0.41	0.63
	Strategic planning ability	0.41	0.000	0.29	0.52	External environment	0.43	0.000	0.32	0.54
						Strategic positioning	0.21	0.000	0.11	0.33
Hospital vision	Constant = 4.20					Constant = 4.20				
	Internal environment	0.44	0.000	0.33	0.55	Quality of care	0.39	0.000	0.28	0.50
	Strategic planning ability	0.34	0.000	0.23	0.45	External environment	0.36	0.000	0.26	0.47
						Strategic positioning	0.18	0.001	0.07	0.28
SMART objectives	Constant =3.62					Constant = 3.62				
	Strategic planning ability	0.56	0.000	0.38	0.74	Strategic positioning	0.63	0.000	0.47	0.80
						External environment	0.23	0.007	0.06	0.39
Action plan	Constant = 3.44					Constant = 3.44				
	Strategic planning ability	0.55	0.000	0.36	0.74	Strategic positioning	0.56	0.000	0.38	0.74
	Internal environment	0.22	0.022	0.03	0.41	External environment	0.33	0.000	0.15	0.51

The four regression models, hospital mission, vision, SMART objectives, and action plan, as outcome dimensions were significant. At the start of the training program, strategic planning ability (PC I-1) and internal environment (PC I-2) had a positive significant influence on the quality of the description of the hospital’s mission, vision, and action plan, while only strategic planning ability (PC I-1) had a positive significant influence on SMART objectives. Twelve months after the end of the training program, the external environment (PC II-1), strategic positioning (PC II-2), and quality of care (PC II-3) had a positive significant influence on the quality of the description of the hospital’s mission and vision. External environment (PC II-1) and strategic positioning (PC II-2) had a positive significant influence on SMART objectives and the action plan (Table 8).

## Discussion

The aim of this study was to determine learning outcomes after a specific training program for hospital management teams. The results of our training program for hospital management teams, offered by the Centre School of Public Health and Management in Belgrade, for the first time in Serbia, provide evidence which support training hospital managerial teams in strategic planning and management. The framing of strategic issues is a critical component of hospital management as it can provide health policy makers with the opportunity to take the initiative.

Тhis study analysed the possibility of transferring management principles to healthcare management, throughout assessment of the learning outcomes in management teams, regarding the quality of SWOT analysis and the development of a Strategic Plan. Multiple linear regression analysis identified four significant models: hospital mission, vision, SMART objectives, and the action plan.

Following the training program, external environment, strategic positioning, and the quality of care were predictors of learning outcomes. The quality of the second SWOT analyses was improved mainly because hospital managers recognized the relevance of the external environment exploration and their position in it, based on evidence (patterns of disease, quality of care, costs), stakeholder analysis (i.e., the private sector and other public hospital services and customers) and financial resources. As a result, the teams were able to better describe market dynamics and to propose specific strategies for hospitals, SMART objectives and the action plan. In relation to the open market, hospitals managerial teams tried to improve their position through delivery of new services and resources.

Quality of care was the most important independent component in the definition of hospital mission and vision. The managerial teams recognized the relevance of customers’ and employees’ perspectives for the improvement of mission and vision statements, capacity to deliver better quality of care, and patient satisfaction enhancement.

SWOT analysis is one of the most common tools in strategic management, e.g. in the Netherlands more than 80% of health managers in hospitals, home care organizations and nursing homes are reported to use the SWOT analysis as part of their strategic process [38]. SWOT, when used properly, can provide a good basis for the formulation of strategy [41]. In our study, terms in the questionnaire, used by reviewers for evaluation of SWOT analysis, could be queried in many ways, but together were able to demonstrate improvement after completion of the training program. Furthermore, external evaluation and review were designed to provide support to hospitals by improving systematic determination of quality, “valued outcomes”, and “key contributing processes” [42]. The reviewer-based evaluation was characterized by a high level of internal consistency.

In Serbia, a transitional country with a socialist heritage and little modern management experience, defining a hospital’s mission, vision, action plan, and especially its SMART objectives, seems to be dependent on the political environment and existing legislation. The measurement and evaluation of hospital performance were recognized as essential, partly because of the recently established reporting system of quality indicators [43] and partly due to recognition of the usefulness for benchmarking. Only a few stakeholders, i.e., the Ministry of Health, the Republic Health Insurance Fund and project agencies were considered relevant for the hospitals services and financial flow. This demonstrated that the managerial teams were predominantly oriented toward the fulfillment of legal obligations and contracts. The hospital’s internal environment (staff, their training and development, management, information system, equipment, customers and their satisfaction, and type and quality of health services) was included in the government’s health reform initiatives [25].

Positive effects of the independent components on learning outcomes, i.e., mission and vision, SMART objectives, and action plan, indicated that the extensive discussions initiated between the members of the managerial teams led to improved operation, even though their approach has not yet been formalized optimally. The teams understood the critical elements of the strategic planning process, but had difficulties in making maximum use of them in concretely planning their activities, e.g. balancing appropriate efficiency and quality [44]. This is obviously a desideratum in the improvement and better focusing of the didactic elements in training programs and their operational transfer.

However, the weakness of our study is the lack of control group, so our results cannot be fully attributed to the training programme. An alternative explanation is that some managers might have had some individual training or previous experience or some of them were interested in deeper research in specific fields. A potential weakness was the composite judgment of “teams”, without singling out individual members of the management teams. This is especially relevant as the composition of the multidisciplinary teams varied to some degree with respect to their professional background, reflecting the specific circumstances of the 20 participating hospitals. However, the chosen team approach is an asset in terms of sustainability of the training effects in transferring advanced managerial competence to several lead persons within a certain hospital. Accordingly, mutual understanding as well as a stronger impact in pursuing the necessary organizational changes in the hospital can be expected.

Research evidence demonstrates that improving hospitals’ strategic planning practices can be effective, but many health care organizations have difficulties in implementing their Strategic Plan to result in successful performance [45,46]. Also, both the hospital’s mission and vision statement and its clearly defined objectives are related to improved performance, staff behavior, and staff motivation [47,48]. This is evidenced by the formal training programs that have been ongoing in some countries, in which it was concluded that the positive effects of those programs justified additional investment in future education and training [49]. In Serbia, this was the first time that communities had the possibility of learning about their hospitals. When the managerial teams presented their strategies and action plans at a public event, this had a positive impact on the recognition of hospitals in their local environment.

The need for stronger management in Serbian health care is well recognized and the European Union, in particular, is supporting management capacity building programs [25-28]. Continuing education in health care management is being offered in Serbia at an increasing scale, in response to the health care system’s well-known deficits. A Masters program in health policy and management was recently established at the Belgrade Faculty of Medicine. Furthermore, a separate Masters in health management was also initiated. However, those programs have not been evaluated.

Through its policy and organization of the health care system, Serbia allows directors of the healthcare institutions to be physicians without formal managerial education. In such cases, introduction of training for the complete managerial team creates the conditions to maximize knowledge transfer in the workplace and encourage action oriented leadership. This is the value of our study for the international audience, since multidisciplinary managerial teamwork leads to success with a strong focus on organizational values, culture and interpersonal relationship.

## Conclusion

In Serbia, for the first time, hospital managers were trained as a team by the Centre School of Public Health and Management in Belgrade to develop an official Strategic Plan for their hospitals and to implement monitoring and adjustment of their strategies. The training program had positive effects on the teams’ abilities to develop their hospital’s mission and vision, strategic objectives, and action plan as learning outcomes.
